# Development of an Efficient Gene Editing Tool in *Schizochytrium* sp. and Improving Its Lipid and Terpenoid Biosynthesis

**DOI:** 10.3389/fnut.2021.795651

**Published:** 2021-12-14

**Authors:** Peng-Wei Huang, Ying-Shuang Xu, Xiao-Man Sun, Tian-Qiong Shi, Yang Gu, Chao Ye, He Huang

**Affiliations:** ^1^School of Food Science and Pharmaceutical Engineering, Nanjing Normal University, Nanjing, China; ^2^College of Biotechnology and Pharmaceutical Engineering, Nanjing Tech University, Nanjing, China

**Keywords:** *Schizochytrium* sp., *Agrobacterium tumefaciens*, genetic manipulation, terpenoids, lipid

## Abstract

*Schizochytrium* sp. HX-308 is a marine microalga with fast growth and high lipid content, which has potential as microbial cell factories for lipid compound biosynthesis. It is significant to develop efficient genetic editing tool and discover molecular target in *Schizochytrium* sp. HX-308 for lipid compound biosynthesis. In this study, we developed an efficient gene editing tool in HX-308 which was mediated by *Agrobacterium tumefaciens* AGL-1. Results showed that the random integration efficiency reached 100%, and the homologous recombination efficiency reached about 30%. Furthermore, the metabolic pathway of lipid and terpenoid biosynthesis were engineered. Firstly, the acetyl-CoA *c*-acetyltransferase was overexpressed in HX-308 with a strong constitutive promoter. With the overexpression of acetyl-CoA c-acetyltransferase, more acetyl-CoA was used to synthesize terpenoids, and the production of squalene, β-carotene and astaxanthin was increased 5.4, 1.8, and 2.4 times, respectively. Interestingly, the production of saturated fatty acids and polyunsaturated fatty acids also changed. Moreover, three Acyl-CoA oxidase genes which catalyze the first step of β-oxidation were knocked out using homologous recombination. Results showed that the production of lipids increased in the three knock-out strains. Our results demonstrated that the *A. tumefaciens*-mediated transformation method will be of great use for the study of function genes, as well as developing *Schizochytrium* sp. as a strong cell factory for producing high value products.

## Introduction

Oleaginous microalgae can accumulate up to 30% of lipids, which are valuable commercial resources (1). These lipids can be divided into two categories: saturated fatty acids with 14–16 carbons can be used as biodiesel fuel; polyunsaturated fatty acids with more than 20 carbons can be used as health care products (2, 3). *Schizochytrium* sp. HX-308 (CCTCC M 209059) is a marine microalga which belongs to thraustochytrids genera, and it was isolated from seawater with fast growth and high lipid yields (4, 5). Previously, our studies have reported that the lipid yield and the cell dry weight (CDW) of HX-308 can reach 80.14 g/L and 134.5 g/L, respectively (1, 5–7). However, manipulation of fermentation conditions to promote the productivity of target products seems to have reached choke point. With the development of molecular biology technologies, genetic engineering is becoming more and more common strategies for improving the production of high value products in microalgae (8–12). Thus, it is of great importance to develop efficient gene editing tools in HX-308 for constructing it as a better cell factory.

It has been reported that electroporation is the main approach to introduce exogenous DNA into *Schizochytrium* (13). However, the efficiency of homologous recombination is very low after electroporation. Sakaguchi et al. tried to knockout the Δ5 desaturase gene in *Thraustochytrium aureum* ATCC 34304 but only destroyed one allele in the first round of electroporation (13). They had to carry a second round of electroporation to knockout the other allele, which cost them more time and money. Apart from electroporation, *Agrobacterium tumefaciens* mediated transformation (ATMT) can also be used to transform DNA into other organisms (14). *Agrobacterium tumefaciens* was widely applied for the transformation of plant cells. *Agrobacterium tumefaciens* can transform the T-DNA on the Ti plasmid into plant cells, and the T-DNA was then integrated into the nuclear chromosomes randomly (15, 16). In recent years, ATMT has also been widely used for the transformation of fungi, including yeasts and filamentous fungi (17–19). In addition, it was reported that ATMT can lead to homologous recombination and thus promote the efficiency of gene knockout (20). Therefore, ATMT may also be a promising approach for efficient gene editing in *Schizochytrium*.

In this study, an efficient gene editing tool in HX-308 was constructed based on ATMT. Firstly, a *NeoR* resistance expression cassette was constructed and transformed into HX-308 successfully. Then, the gene acetyl-CoA *c*-acetyltransferase (*AACT*) was overexpressed under the control of the promoter of the endogenous gene acetyl-CoA carboxylase (*ACCase*), and more acetyl-CoA was pulled into the mevalonate (MVA) pathway to improve the productivity of terpenoids. Furthermore, three genes associated with β-oxidation which were named acyl-CoA oxidases (*Acox*) were knocked out using homologous recombination to improve total fatty acids. This study would facilitate the genetic engineering of other thraustochytrids for the production of more high value products.

## Materials and Methods

### Strains and Culture Media

*Schizochytrium* sp. HX-308 (CCTCC M 209059) is a microalga which was isolated from seawater and stored in the China Center for Type Culture Collection (CCTCC) (1). The strain was stored at −80°C in 20% (v/v) glycerol. The seed culture medium and batch culture medium were the same as those described in our previous study (5). The seed of HX-308 was cultured for three passages and then 10 mL of the seed was transferred to 500-mL shake flasks containing 100 mL of medium for batch culture. HX-308 was cultured at 28°C with a rotation speed of 170 rpm. The strain *A. tumefaciens* AGL-1 and the plasmid pZPK were kind gifts from Dr. Sheng Yang (CAS Center for Excellence in Molecular Plant Science).

### Plasmid Construction

**Figure 1:**
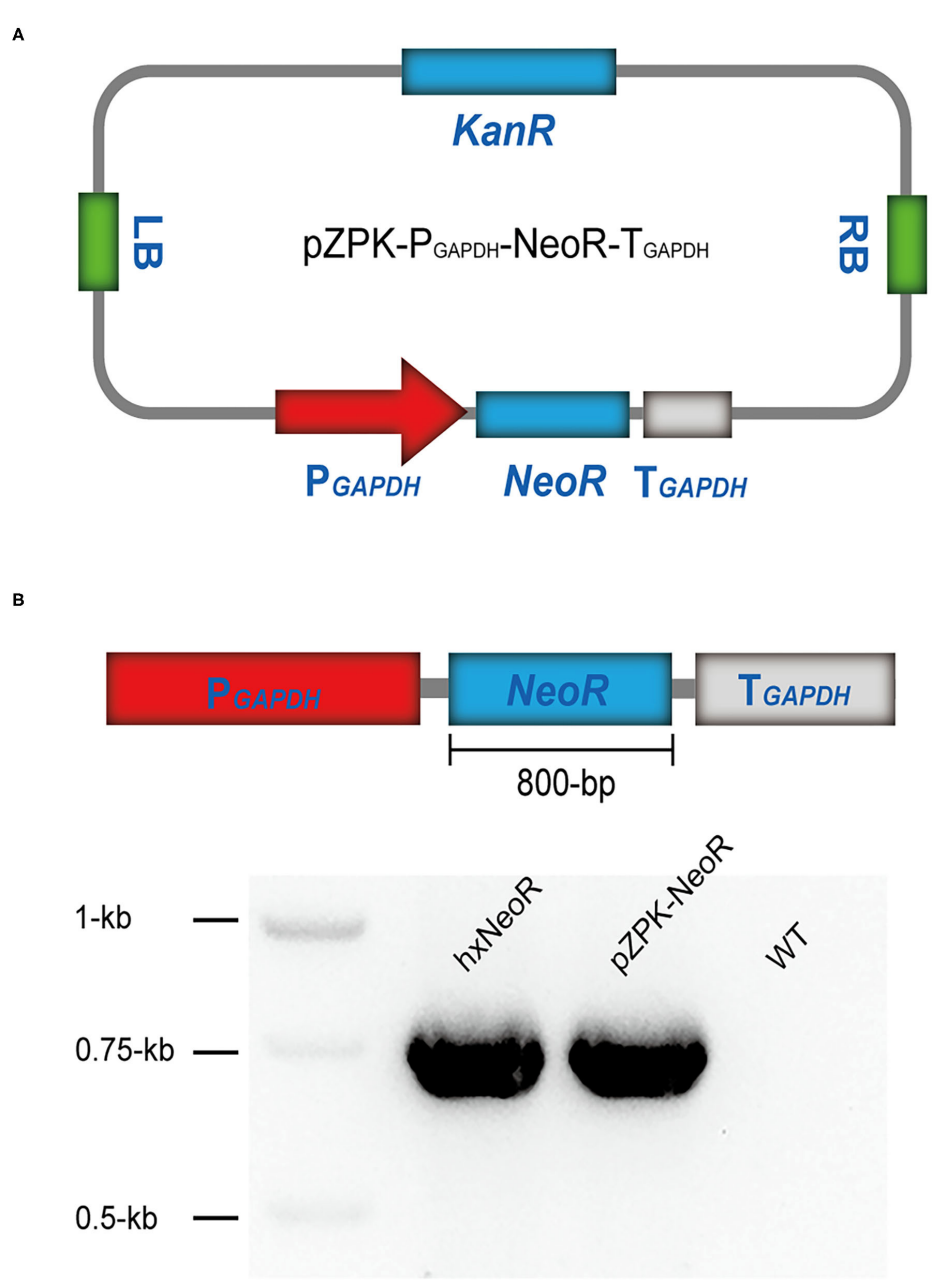
The construction of the plasmid pZPK-P_*GAPDH*_-NeoR-T_*GAPDH*_ and the transformation of HX-308. **(A)** Map of the plasmid pZPK-P_*GAPDH*_-NeoR-T_*GAPDH*_. **(B)** The schematic diagram of the *NeoR* expression cassette and diagnostic PCR validation of the *NeoR* gene.

**Figure 2:**
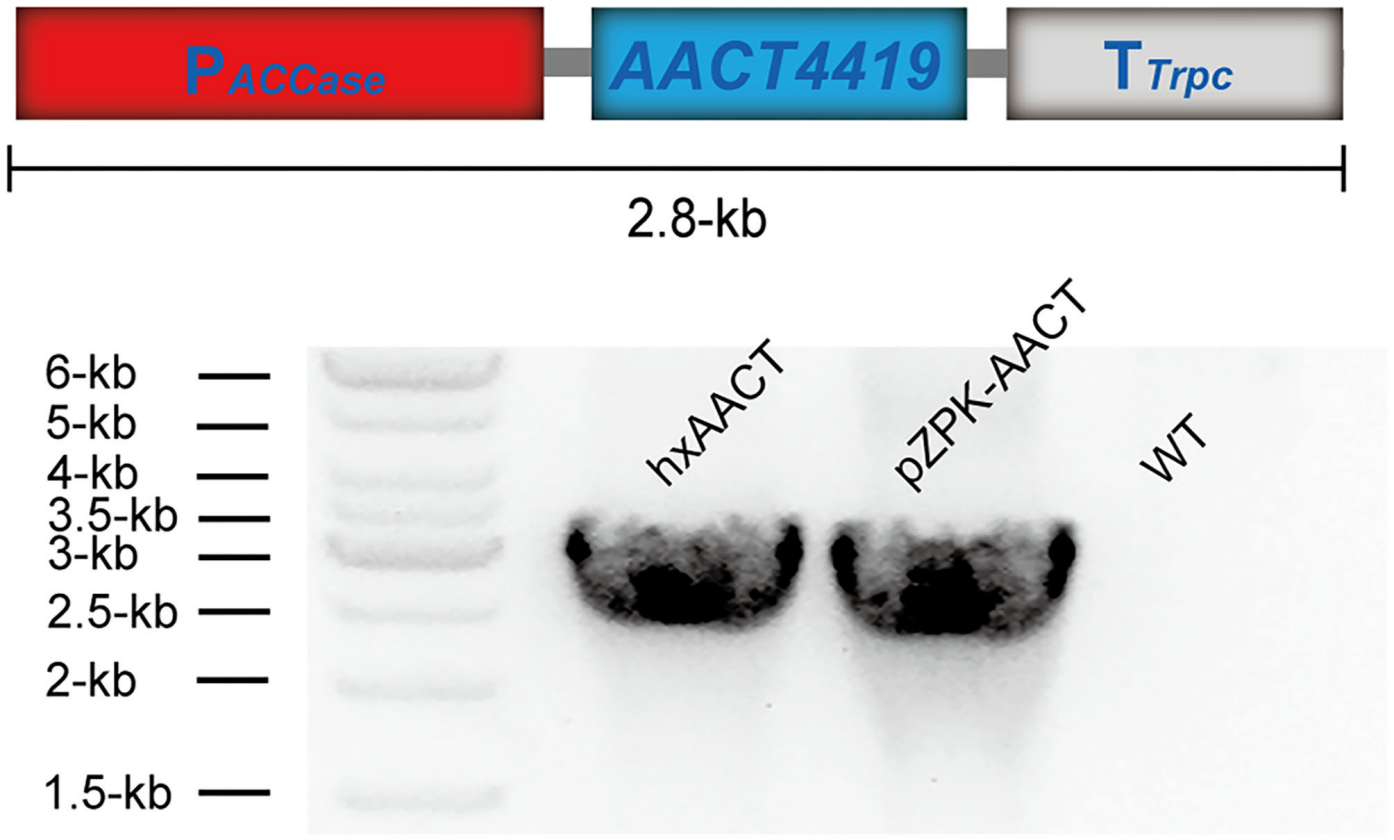
The schematic diagram of the *AACT4419* expression cassette and the diagnostic PCR validation of the *AACT4419* expression cassette.

A binary vector named pZPK was used to carry the DNA elements which were used in this study (21). The vector pZPK-P_*GAPDH*_-NeoR-T_*GAPDH*_ was constructed by inserting a 2.3-kb neomycin resistance (*NeoR*) gene expression cassette into the *EcoRI-XbaI* site of pZPK. The 2.3-kb *NeoR* resistance cassette contained a 1-kb promoter of the endogenous gene glyceraldehyde-3-phosphate dehydrogenase (*GAPDH*), a 0.8-kb *NeoR* gene, and a 0.5-kb terminator of *GAPDH* (Figure 1A). The plasmid pZPK-AACT was constructed by inserting an *AACT* expression cassette into the *HindIII* site of pZPK-P_*GAPDH*_-NeoR-T_*GAPDH*_. The *AACT* expression cassette contained a 1-kb promoter of the endogenous gene acetyl-CoA carboxylase (*ACCase*), the gene *AACT*, and the *Trpc* terminator (Figure 2). The plasmid pZPK-Acox1 was constructed by inserting a 4.3-kb targeted knock-out sequence into the *EcoRI-XbaI* site of pZPK. The sequence contained a 1-kb region in the upstream of the *acox1*, the *NeoR* resistance cassette, and a 1-kb region in the downstream of the *acox1*. The vector pZPK-Acox2 and pZPK-Acox3 were constructed the same way as pZPK-Acox1. The primers described above were listed in Supplementary Table S1.

### The Transformation of HX-308 Mediated by *A. tumefaciens* AGL-1

The transformation method of HX-308 used was a modification of that of Cheng et al. (20). HX-308 was cultured overnight in seed medium, and then was inoculated to fresh seed medium and grown to a logarithmic phase. Cells were harvested by centrifugation at 1000g for 5 min, and then were washed with sterile water for two times. The *A. tumefaciens* harboring plasmids was cultured at 28°C with a rotation speed of 220 rpm overnight in YEB medium supplemented with 50 μg/mL of kanamycin (pH 7.5). Cells were harvested by centrifugation at 4000g for 5 min, and then were resuspended in liquid induction medium (IM) supplemented with 50 μg/mL of kanamycin and 200 μM acetosyringone (pH 5.4). After 8 h of pre-induction at 28°C with a rotation speed of 220 rpm, *A. tumefaciens* was harvested by centrifugation at 4000g for 5 min, and then was washed with sterile water for two times. For transformation, 1 × 10^7^ HX-308 cells and 1 × 10^8^ *A. tumefaciens* cells were mixed and then were spread on IM plates supplemented with 50 μg/mL of kanamycin and 200 μM acetosyringone (pH 5.4). After 48 h of induction at 28°C, the cocultures were washed with sterile water and then were selected on GPYS (10 g yeast extract, 20 g tryptone, 20 g sea life, 50 g glucose, 20 g agar per liter) plates supplemented with 300 μg/mL of cefotaxime sodium and 500 μg/mL of G418 at 28°C for three to five days.

### PCR Analysis of the Transformants

The transformants were cultured for at least two passages before PCR analysis. The genomic DNA of the transformants were isolated. Primer sets NeoR-test-F/R were used to verify the incorporation of the NeoR resistance gene. Primer sets P2520-F and Trpc-R were used to verify the incorporation of the *AACT* gene. Primer sets ACOX1-test1-F/R, ACOX1-test2-F/R, ACOX2-test1-F/R, ACOX2-test2-F/R, ACOX3-test1-F/R, and ACOX3-test2-F/R were used to verify the disruption of the *ACOX1, ACOX2*, and *ACOX3* gene. The primers described above were listed in Supplementary Table S2.

### Determination of Biomass, Glucose, and Lipid Production

To measure CDW, 10 mL of the culture was harvested by centrifugation at 5000g for 5 min. The sediments were dried at 60°C for 48 h to constant weight, and was then used for gravimetric analysis. For extracellular glucose and the production of total lipids, they were measured according to our previous study (4, 5).

### Fatty Acids and Terpenoids Analysis

To determine fatty acids, 0.1 g of biomass was used to prepare fatty acid methyl esters (FAMEs). The preparation methods used were according to our previous study (4). And the FAMEs were then analyzed using the gas chromatography system (GC-2014; Shimadzu, Japan) equipped with a 60 m × 0.22 mm capillary column (DB-23; Agilent, USA) and a flame ionization detector (FID). The carrier gas was nitrogen. The detection methods used were according to our previous study (5). Authentic FAMEs reference standards were purchased from Sigma-Aldric, USA.

To determine terpenoids, one gram of biomass was firstly freeze-dried at −20°C for 48 h. The residue was ground at 60 Hz, and then be extracted with 1 mL of hexane. β-carotene and astaxanthin contents were quantitated using the high performance liquid chromatography (1260 Infinity II; Agilent, USA) equipped with a 4.6 mm × 150 mm column (Eclipse XDB-C18; Agilent, USA). The analytical condition was a modification of that of Harnkarnsujarit et al. (22). A gradient solvent system of MeCN/H_2_O (90/10, v/v) (solvent A) and MeOH/IPA (60/40, v/v) (solvent B) were used: 100% solvent A and 0% solvent B was used firstly, then solvent A was decreased to 10% and solvent B was increased to 90% within 15 min and retained from 15 to 30 min. The detection wavelength was 470 nm, and the flow rate was 1.0 mL/min. Authentic β-carotene and astaxanthin reference standards were purchased from Sigma-Aldric, USA.

### Statistical Analysis

This study carried out analyses on the glucose consumption, CDW, total fatty acids, squalene productivity, β-carotene productivity, astaxanthin productivity and the percentage of fatty acids. The analyses were performed by GraphPad Prism 8, and the results of the quantification were presented as the mean value ± standard deviation from at least three replicates.

## Results and Discussion

### Optimization of the Selection Condition of *Schizochytrium* sp. HX-308 and the Construction of G418 Resistant Expression Cassette

It was reported that G418 could inhibit the growth of *Schizochytrium* sp. strain SEK 579 (13). However, the inhibition data of G418 to the strain HX-308 was unknown. Thus, the sensitivity of HX-308 to G418 was determined and then been used to select transformants. The results appeared that 300 μg/mL or higher concentration of G418 was able to inhibit the growth of HX-308 on GPYS solid plates (Supplementary Figure S1). Thus, to avoid false positive results, GPYS solid plates supplemented with 500 μg/mL of G418 and 300 μg/mL of cefotaxime sodium were used to select HX-308 transformants in the next studies. The cefotaxime sodium was used to kill *A. tumefaciens* which was used to transform DNA into HX-308 after transformation (23).

The gene *NeoR* encodes the enzyme neomycin phosphotransferase, which could confer resistance to the aminoglycoside antibiotic G418 in HX-308 transformants (24). Thus, a *NeoR* expression cassette should be constructed under the control of a promising promoter. The promoter of a housekeeping gene *GAPDH* which expresses constantly was chosen as a candidate (25). The coding sequence of the endogenous gene *GAPDH* in HX-308 was found according to the annotation of the genome sequence. The 1-kb promoter of *GAPDH* (P_*GAPDH*_) in HX-308 was cloned and been used to drive the expression of the gene *NeoR*. Thus, the *NeoR* expression cassette driven by P_*GAPDH*_ was inserted into the vector pZPK, resulting in a new vector pZPK-P_*GAPDH*_-NeoR-T_*GAPDH*_ (Figure 1A). Luckily, the results in the next studies appeared that the promoter P_*GAPDH*_ worked successfully in HX-308. Some other promoters such as P_*TEF*−1_, P_*AOX*1_, and P_*EF*−1_ were also reported to work in *Schizochytrium* sp. ATCC 20888 (8). However, the promoters were not the endogenous promoters, expression intensity maybe not be enough in HX-308. Thus, more promoters should be characterized by the β-Galactosidase reporter system or the EGFP reporter system in the future (8).

### Transformation of *Schizochytrium* sp. HX-308 Mediated With *A. tumefaciens* AGL-1

The *A. tumefaciens* AGL-1 containing the plasmid pZPK-P_*GAPDH*_-NeoR-T_*GAPDH*_ was used to transform the *NeoR* expression cassette into HX-308. In a previous study, Cheng et al. transformed an *egfp* gene into *Schizochytrium* and green fluorescence was observed successfully (20). To improve the contact between *Agrobacterium* and *Schizochytrium*, they treated the *Schizochytrium* with DTT and lysing enzymes to weaken the cell wall (20). In this study, we abolished the pretreatment of HX-308 to simplify the transformation procedures. *Agrobacterium tumefaciens* AGL-1 were mixed with HX-308 and spread on IM plates which were supplemented with 200 μM acetosyringone. Acetosyringone was used to induce *A. tumefaciens* AGL-1 to integrate the T-DNA containing the *NeoR* expression cassette into the HX-308 genome (26). After induction, the cocultures were selected on GPYS plates supplemented with cefotaxime sodium and G418. The results appeared that *A. tumefaciens* AGL-1 could also attach to HX-308 even without weakening the cell wall of *Schizochytrium*.

The colonies were grown on GPYS plates supplemented with cefotaxime sodium and G418 for at least three generations, and the transformants were then used to extract genomic DNA for PCR analysis. The insertion of the *NeoR* gene in the transformants was confirmed by diagnostic PCR. The transformants generated an expected ~800-bp band, but the *NeoR* gene could not be detected in the wild type (WT) (Figure 1B), indicating that the *NeoR* gene was integrated into the genome of HX-308. Our results demonstrated that the ATMT method we developed was a promising strategy to transform HX-308.

### Overexpression of the Acetyl-CoA *c*-Acetyltransferase Gene in *Schizochytrium* sp. HX-308 for Enhancing the Terpenoid Biosynthesis

The successful expression of the *NeoR* gene suggested that the ATMT method can be used to overexpress functional genes in HX-308. In our previous study, HX-308 can produce over 80 g/L lipid during fed-batch culture, which indicated that it can produce high level of acetyl-CoA as the precursor for lipid biosynthesis (5, 27). Similarly, acetyl-CoA is also the precursor of terpenoids. And it was reported that *Schizochytrium sp*. could synthesize some terpenoids such as squalene, β-carotene and astaxanthin during fermentation (28–30). However, the productivity of terpenoids in *Schizochytrium sp*. was quite low. In recent years, genetic engineering strategies are commonly being used to increase terpenoids production in microalgae (31). Thus, it would be hopeful to pull some acetyl-CoA from the biosynthesis of lipids to enhance the biosynthesis of these valuable terpenoids in HX-308 through genetic manipulation. In nature, all terpenoids can be synthesized via the MVA pathway or the methylerythritol phosphate (MEP) pathway, and HX-308 utilizes the MVA pathway to synthesize terpenoids. In this study, the gene acetyl-CoA c-acetyltransferase (*AACT4419*) was overexpressed to increase terpenoid productivity in HX-308. It catalyzes the condensation of two molecules of acetyl-CoA, and a molecule of acetoacetyl-CoA is formed (31). This is the first step of the MVA pathway. The strong promoter P_*ACCase*_ was chosen to drive the expression of the gene *AACT4419*, resulting the strain hxAACT (Figure 2). ACCase is a constitutive enzyme which catalyzes the carboxylation of acetyl-CoA during the biosynthesis of fatty acids in *Schizochytrium* (8). Thus, the promoter of the gene *ACCase*, P_*ACCase*_, would be a promising promoter to express the gene *AACT4419*, and thus synthesizing more acetoacetyl-CoA to synthesize terpenoids in the strain hxAACT.

**Figure 3:**
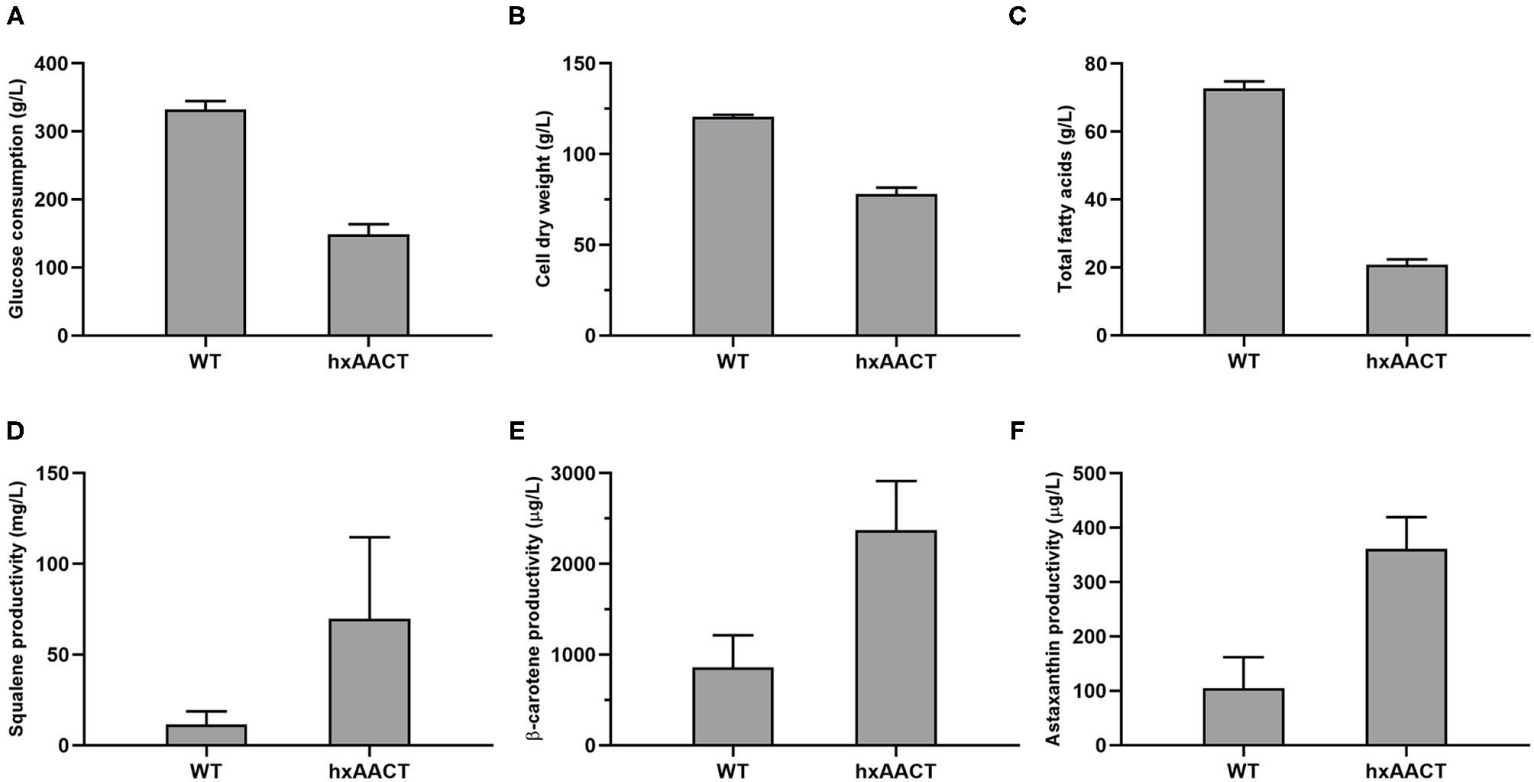
Comparison of cell growth, the accumulation of lipids, and the synthesis of terpenoids between the strain WT and the strain hxAACT at 120 h in 500-mL shake flasks. **(A)** Glucose consumption, **(B)** CDW, **(C)** Lipid productivity, **(D)** Squalene productivity, **(E)** β-carotene productivity, **(F)** Astaxanthin productivity. Values and error bars represent the means and standard deviations from triplicate experiments.

The strains hxAACT4419 and WT were subjected to fed-batch culture for 120 h. What surprised us was that the growth and glucose consumption of hxAACT decreased a lot when compared with WT. By the end of fermentation, the glucose consumption of hxAACT was 148.5 g/L, which was 55% lower than that of the WT (Figure 3A). And the total fatty acids and CDW of hxAACT decreased 71% and 35% respectively when compared with WT (Figures 3B,C). Moreover, as shown in Table 1, the lipid composition of hxAACT also changed significantly. The percentage of 14:0 and docosapentaenoic acid (DPA) remained higher in hxAACT than that in WT throughout the fermentation. The highest percentage of 14:0 was obtained at 72 h in hxAACT, which was 15% higher than that in WT. Similarly, the percentage of DPA in hxAACT remained around 20%, which was 4 to 5% higher than that in WT. In contrast to 14:0 and DPA, the percentage of 16:0 and DHA remained lower in hxAACT than that in WT throughout the fermentation. The percentage of DHA in WT remained around 45%. However, the percentage of DHA in hxAACT decreased rapidly from 42.87 to 20.47% during 24–48 h, and then increased from 20.47 to around 33% during 72–120 h.

**Table 1:** The percentage of fatty acids (w/w%) in WT and hxAACT at 24, 48, 72, 96, 120 h in 500-mL shake flasks.

**Time**	**Strains**	**C14:0**	**C16:0**	**EPA**	**DPA**	**DHA**
24 h	WT	11.24 ± 1.11	26.8 ± 0.99	1.66 ± 0.08	14.27 ± 0.22	44.84 ± 1.80
	hxAACT	18.91 ± 2.01	13.96 ± 0.25	0.93 ± 0.89	18.77 ± 0.87	42.87 ± 2.53
48 h	WT	12.67 ± 0.59	25.06 ± 1.15	1.27 ± 0.06	15.06 ± 0.40	44.58 ± 1.97
	hxAACT	25.54 ± 0.83	14.63 ± 0.26	0.75 ± 0.22	20.47 ± 0.37	20.47 ± 0.37
72 h	WT	10.95 ± 0.08	22.55 ± 1.35	1.07 ± 0.08	15.99 ± 0.06	45.00 ± 1.06
	hxAACT	25.95 ± 1.19	12.60 ± 0.21	1.01 ± 0.14	19.06 ± 0.78	32.88 ± 1.15
96 h	WT	9.49 ± 0.24	20.75 ± 1.41	0.90 ± 0.00	16.84 ± 0.38	46.61 ± 1.30
	hxAACT	23.15 ± 1.11	12.04 ± 0.76	0.71 ± 0.09	21.22 ± 1.37	34.40 ± 1.54
120 h	WT	9.58 ± 0.12	22.36 ± 0.53	0.79 ± 0.04	16.98 ± 0.20	44.45 ± 0.35
	hxAACT	22.61 ± 1.19	11.99 ± 0.30	0.77 ± 0.03	21.13 ± 0.54	33.81 ± 1.03

*Values and error represent the means and standard deviations from triplicate experiments*.

We speculated that the integration site of foreign genes may affect the growth phenotype of HX-308 (Figure 3). Thus, inverse PCR was performed to find out the integration site of the *AACT4419* expression cassette (32). The PCR reagent was sent to be sequenced, and the *AACT4419* expression cassette was found to be integrated about 1.2-kb downstream in the coding sequence of the gene which was named *A3018*, resulting in insertional inactivation of the gene *A3018* (Supplementary Figure S2). The amino acid sequences of the gene *A3018* was then submitted to NCBI for basic local alignment. The results suggested that the gene *A3018* encoded the enzyme UDP-D-xylose: ribitol-5-phosphate beta1,4-xylosyltransferase (TMEM5). TMEM5 was reported to be critical for the biosynthesis of *O*-mannosyl glycan, which is responsible for the biosynthesis of cell wall (33). Further studies can be conducted to knockout the gene *TMEM5* to verify the effect of the gene *A3018* on the growth of HX-308 in the future.

The overexpression of *AACT4419* was aimed to improve the biosynthesis of terpenoids. Thus, the productivity of these three valuable terpenoids in HX-308 were detected. As was expected, the productivity of squalene in the strain hxAACT was increased from 11 mg/L to 70 mg/L, representing a 5.4-fold increase than that of the WT (Figure 3D). Similarly, with the overexpression of the gene *AACT4419* in hxAACT, the production of β-carotene and astaxanthin were increased 1.8 times and 2.4 times, respectively (Figures 3E,F). These findings indicated that the overexpression of *AACT4419* increased terpenoids production in hxAACT, probably by increasing the supply of acetyl-CoA and acetoacetyl-CoA.

### Disruption of *Acox1-3* in HX-308 for Enhancing the Lipid Biosynthesis

**Figure 4:**
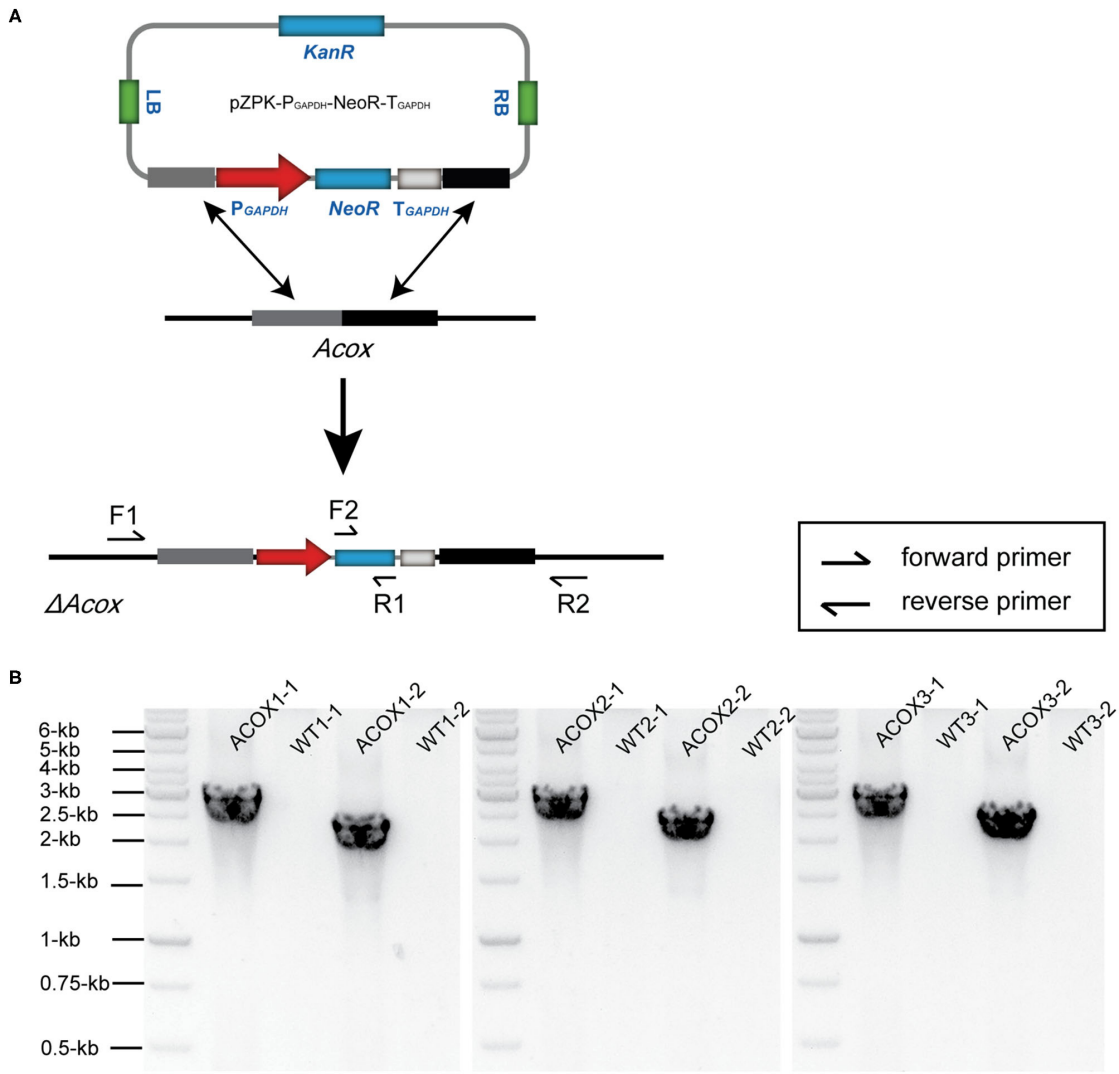
Disruption of the genes *Acox1-3* in HX-308. **(A)** The schematic diagram of the disruption of the genes *Acox1-3*. **(B)** Diagnostic PCR confirmation of the mutants ACOX1, ACOX2, and ACOX3.

β-Oxidation is the process of the metabolism of fatty acids which takes place in both mitochondria and peroxisomes. Thus, the inhibition of β-oxidation may facilitate the accumulation of lipids (34). It was reported that disrupting the gene associated with β-oxidation (*Acox*) could reduce the degradation of fatty acids, and thus increased the accumulation of total lipids in *Aurantiochytrium* sp. (10). Acox catalyzes the transformation of acyl-CoA into trans-2, 3-dehydroacyl-CoA (35). It is the first step of β-oxidation in peroxisome. Thus, knocking out of *Acox* could break the process of β-oxidation, resulting in lipid accumulation. In this study, we identified and knocked out the three *Acox* genes in HX-308 in order to increase the lipid biosynthesis. Three plasmids named pZPK-Acox1-3 were constructed based on the sequences of *Acox1-3*, and then were transformed into HX-308 respectively. During diagnostic PCR, two primer pairs F1/R1 and F2/R2 were used to screen the genome edited transformants of each gene. The primers F1 and R2 were located in the genome of HX-308, outside of the homologous arms of the plasmids pZPK-Acox1-3. The primers F2 and R1 were located in the coding sequence of *NeoR* on the plasmids pZPK-Acox1-3. The mutants generated an expected ~2.9-kb band and an expected 2.4-kb band, but no bands could be generated when WT was used as templates (Figure 4). The results demonstrated that the genes *Acox1-3* were disrupted successfully, and the strains were named hxACOX1, hxACOX2 and hxACOX3. During verification, what surprised us was that the knock-out efficiency of *Acox1, Acox2*, and *Acox3* was about 20% (2 mutants in 10 transfromants), 30% (3 mutants in 10 transfromants), and 16.7% (2 mutants in 12 transfromants) respectively. It was much higher than that in other fungi such *Aspergillus sojae* and *Aspergillus oryzae* with an efficiency of ~5% (36). A previous study also reported that ATMT lead to homologous recombination and facilitated gene knock-out in *Aspergillus carbonarius* (37). Thus, the ATMT method we constructed would be promising for gene disruption in HX-308.

**Figure 5:**
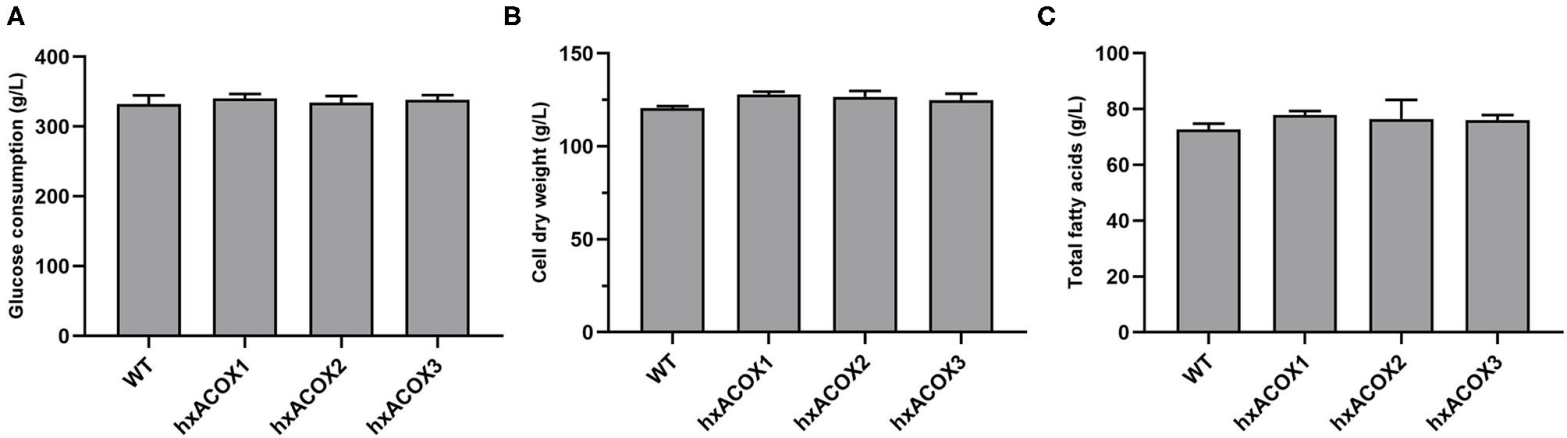
Comparison of cell growth and the accumulation of lipids between the strain WT and the strains ACOX1-3 at 120 h in 500-mL shake flasks. **(A)** Glucose consumption, **(B)** CDW, **(C)** Lipid productivity. Values and error bars represent the means and standard deviations from triplicate experiments.

The strains hxACOX1, hxACOX2, hxACOX3, and WT were subjected to fed-batch culture for 120 h. By the end of fermentation, the glucose consumption of the four strains has no significant difference (Figure 5A). However, the CDW of the strains hxACOX1-3 increased about 6.1, 5.1, and 3.7% when compared with WT (Figure 5B). Accordingly, the total fatty acids of the strains hxACOX1-3 increased about 7.1, 5.1, and 4.7% respectively (Figure 5C). The results appeared that the disruption of *Acox1-3* inhibited the process of β-oxidation, and thus increased the biosynthesis of lipids in HX-308. The Acox1-3 were three isoenzymes which were expressed by HX-308. The disruption of one *Acox* gene can be complemented by other *Acox* genes (38). To further improve the accumulation of lipids in HX-308, the cre-*loxP* system can be constructed in the future to disrupt *Acox1, Acox2*, and *Acox3* simultaneously (39).

## Conclusion

In conclusion, we have firstly established an efficient ATMT method for the genetic manipulation in the *Schizochytrium* sp. HX-308. The gene *AACT4419* was overexpressed and the genes *Acox1-3* were disrupted in HX-308 to improve the productivity of terpenoids and lipids. A strong constitutive promoter P_*ACCase*_ was used to drive the expression of *AACT4419*, and the squalene, β-carotene and astaxanthin yields of the resulting strain hxAACT were increased 5.4, 1.8 and 2.4 times compared to the WT. When disrupting the genes associated with β-oxidation, the resulting strains hxACOX1, hxACOX2, and hxACOX3 reached a CDW of 127.9, 126.7, and 124.9 g/L. And the lipid yields of hxACOX1, hxACOX2, and hxACOX3 were increased 7.1, 5.1, and 4.7% respectively. In the future, the ATMT method we constructed would be beneficial to studying functional genes as well as producing high value products in HX-308.

## Nucleotide Sequence Accession Numbers

The accession numbers of the sequences reported in this article deposited in GenBank: OK641580 (*GAPDH*), OK641581 (*AACT*), OK641582 (*ACOX1*), OK641583 (*ACOX2*), OK641584 (*ACOX3*), OK641585 (*ACCase*).

## Data Availability Statement

The original contributions presented in the study are included in the article/Supplementary Material, further inquiries can be directed to the corresponding author.

## Author Contributions

P-WH carried out the experiments and drafted the manuscript. Y-SX and X-MS analyzed the data and helped to draft the manuscript. T-QS, YG, CY, and HH conceived and designed the study and revised the manuscript. All authors read and approved the final manuscript.

## Funding

This work was supported by the Nature Science Foundation of Jiangsu Province (No. BK20190706), the National Natural Science Foundation of China (Nos. 21908112 and 22038007).

## Conflict of Interest

The authors declare that the research was conducted in the absence of any commercial or financial relationships that could be construed as a potential conflict of interest.

## Publisher's Note

All claims expressed in this article are solely those of the authors and do not necessarily represent those of their affiliated organizations, or those of the publisher, the editors and the reviewers. Any product that may be evaluated in this article, or claim that may be made by its manufacturer, is not guaranteed or endorsed by the publisher.
