# Supplementary material for: Development of an Efficient Gene Editing Tool in Schizochytrium sp. and Improving Its Lipid and Terpenoid Biosynthesis
Source: Front Nutr. 2021 Dec 14;8:795651. doi: 10.3389/fnut.2021.795651 (PMC8712325; doi:10.3389/fnut.2021.795651)
Supplement: Supplementary file 1 [file Data_Sheet_1.doc]

**Table S1. Primers used for plasmid construction in this study.**

| Name | Sequence |
| --- | --- |
| P_GAPDH_-F | AATTAACGCCGAATTGAATTTTTCTCGACACTTGTCTCCG |
| P_GAPDH_-R | AATCCATCTTGTTCAATCATCTTTTCTCTCGCCTCTCGCT |
| NeoR-F | AGCGAGAGGCGAGAGAAAAGATGATTGAACAAGATGGATT |
| NeoR-R | AAAAGCTCGCGTGCGACTTTTCAGAAGAACTCGTCAAGAA |
| T_GAPDH_-F | TTCTTGACGAGTTCTTCTGAAAAGTCGCACGCGAGCTTTT |
| T_GAPDH_-R | TGCTGCAGGTCGACTCTAGACGATAAGGCTACCCTAGAGC |
| P_ACCase_-F | GATCCAAGCTCAAGCTGCCAGCAACCAAAGCAACCAGAGC |
| P_ACCase_ -R | AACGTTAAGTGaagcttTGTTCCTGCTGCTGCT |
| AACT4419-F | AGCAGCAGCAGCAGGAACAaATGTCCAAGATGGAGACTATCC |
| AACT4419-R | ATTTCAGTAACGTTAAGTGaTTAGGCGAGGCGCTCCAC |
| Trpc-F | AGGAACAaagcttCACTTAACGTTACTGAAATC |
| Trpc-R | GAGTCGACCTGCAGCATGCAGATTTCGAGGTTTATACCTA |
| ACOX1-Left-F | ACTGAATTAACGCCGAATTGCGAGAGCGAGAACAGGCTAG |
| ACOX1-Left-R | CGGAGACAAGTGTCGAGAAAAAAGCGAGTGCAAGATTGCG |
| ACOX1-Right-F | GCTCTAGGGTAGCCTTATCGtctagaaTCACAAGCAACTGGCTCACC |
| ACOX1-Right-R | TTGCATGCTGCAGGTCGACTGATGGGTCGGGCTTATTGTG |
| ACOX2-Left-F | ACTGAATTAACGCCGAATTGCGCAGGTTCATTATGATGCC |
| ACOX2-Left-R | CGGAGACAAGTGTCGAGAAAGCCTCATTCATCCAGCTTCC |
| ACOX2-Right-F | GCTCTAGGGTAGCCTTATCGtctagaCGTGGCTAACAAATCTGACG |
| ACOX2-Right-R | TTGCATGCTGCAGGTCGACTTAAAGTTTGGCATCACGCTG |
| ACOX3-Left-F | ACTGAATTAACGCCGAATTGGAGCTCCGAAGATCTGCAGG |
| ACOX3-Left-R | CGGAGACAAGTGTCGAGAAATCATCTTGCGCGTATCCCTG |
| ACOX3-Right-F | GCTCTAGGGTAGCCTTATCGtctagaGCTTCCGTCTGCTCAAGTGG |
| ACOX3-Right-R | TTGCATGCTGCAGGTCGACTTTTGGCCGAAGTGACAGCAG |
| P_GAPDH_-HindIII-F | TTTCTCGACACTTGTCTCCG |
| T_GAPDH_ -HindIII-R | CGATAAGGCTACCCTAGAGC |

**Table S2. Primers for diagnostic PCR** in this study.

| Name | Sequence |
| --- | --- |
| NeoR-test-F | TGATTGAACAAGATGGATTG |
| NeoR-test-R | CAGAAGAACTCGTCAAGAAG |
| P2520-F | GCAACCAAAGCAACCAGAGC |
| Trpc-R | GATTTCGAGGTTTATACCTA |
| ACOX1-test1-F | TGGACCTGGAGTCCGAGAAG |
| ACOX1-test1-R | CAGAAGAACTCGTCAAGAAG |
| ACOX1-test2-F | TGATTGAACAAGATGGATTG |
| ACOX1-test2-R | GGTACGAAGAGGTATCATCC |
| ACOX2-test1-F | GCACATGCTGCCTATGGAG |
| ACOX2-test1-R | CAGAAGAACTCGTCAAGAAG |
| ACOX2-test2-F | TGATTGAACAAGATGGATTG |
| ACOX2-test2-R | AGAGAGCCGAGGCTACTAC |
| ACOX3-test1-F | GCAGACCTCGAAGACCAGG |
| ACOX3-test1-R | CAGAAGAACTCGTCAAGAAG |
| ACOX3-test2-F | TGATTGAACAAGATGGATTG |
| ACOX3-test2-R | GCTCTGAGTCTTCAAGCTG |


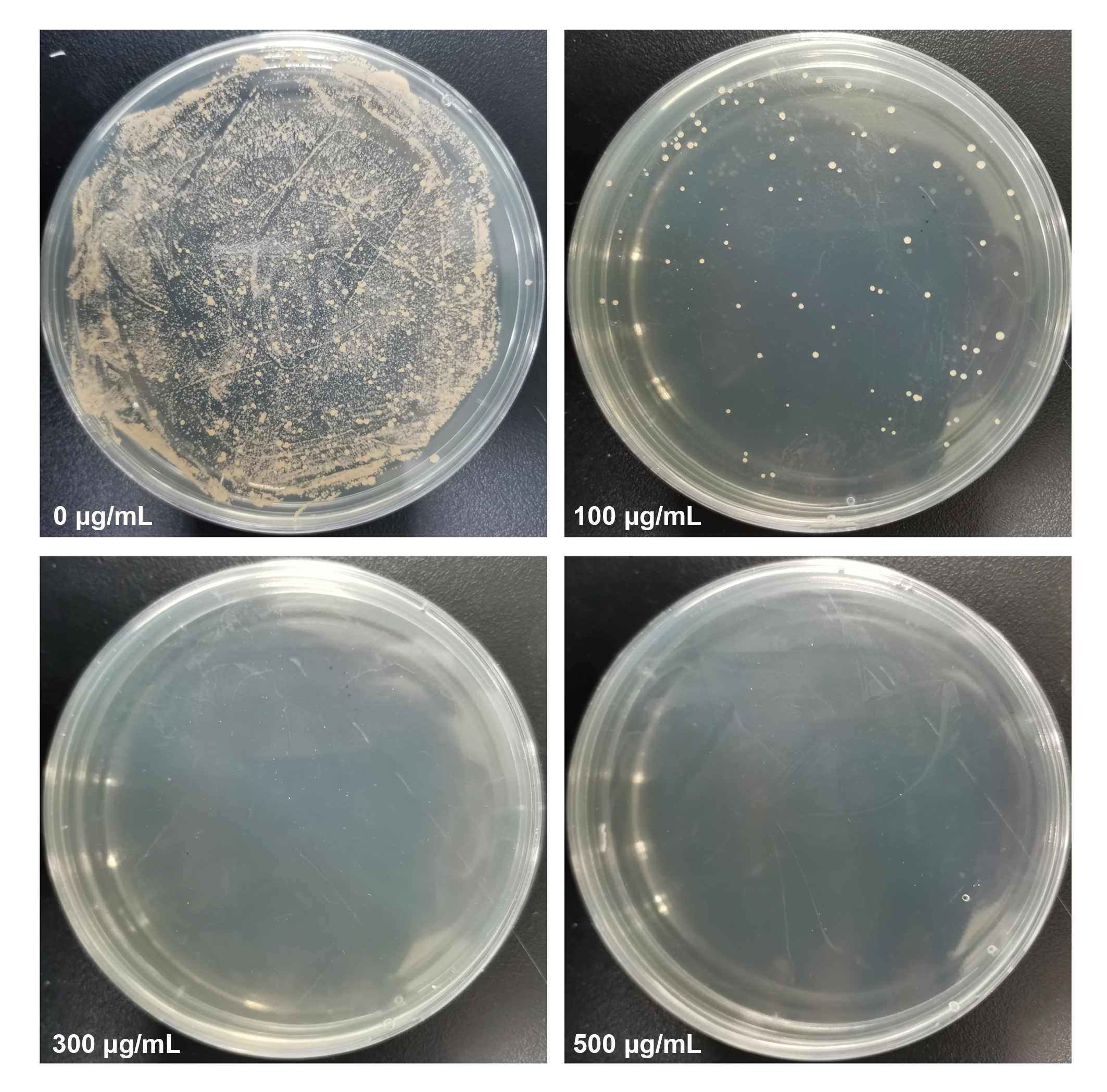


**Figure S1. The growth of *Schizochytrium* sp. HX-308 on GPYS plates supplemented with 0, 100, 300, and 500 μg/mL of G418.**


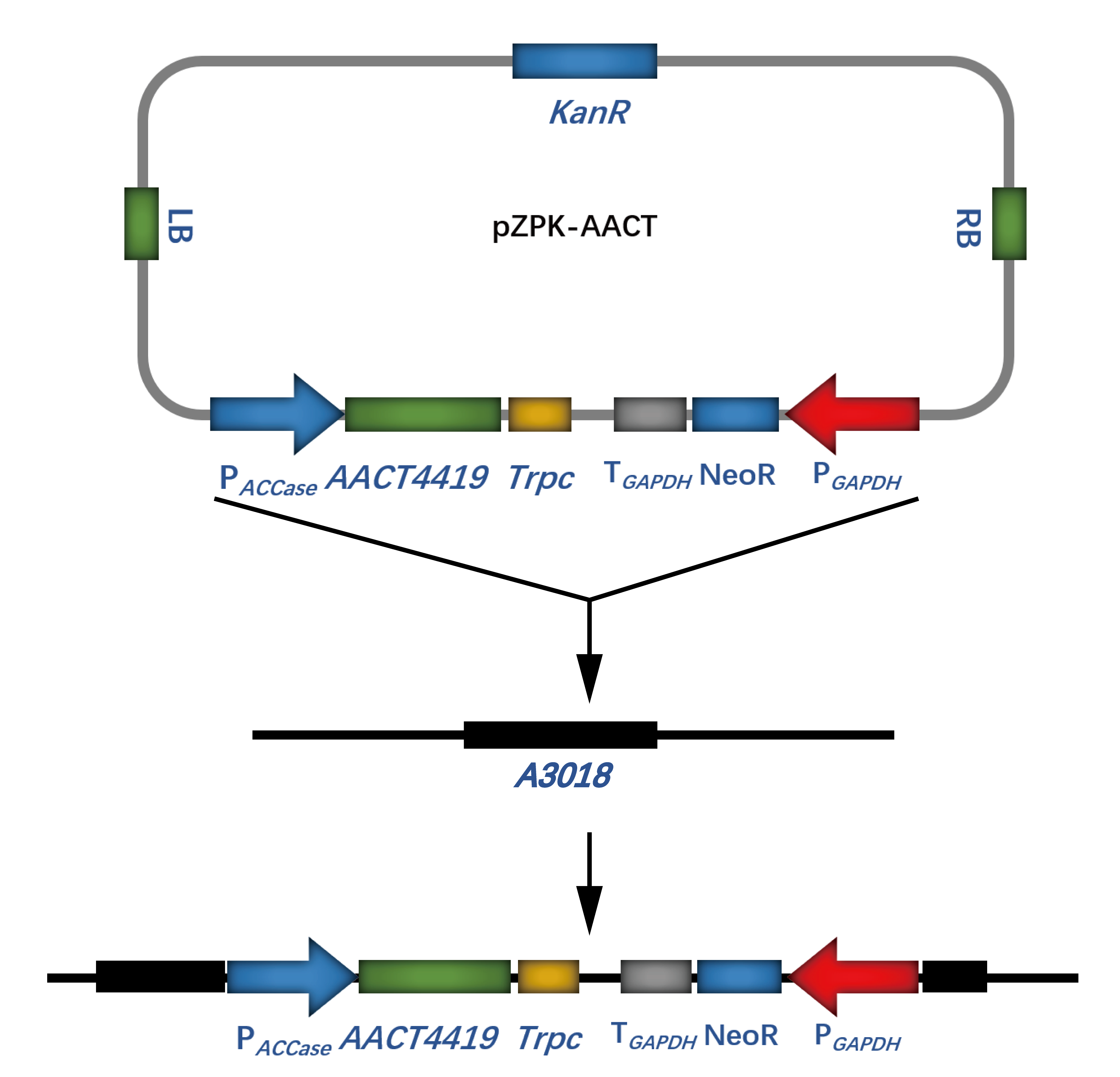


**Figure S2. The schematic diagram of the insertion of the *AACT4419* expression cassette and the *NeoR* expression cassette. The *AACT4419* expression cassette was integrated about 1.2-kb downstream in the coding sequence of the gene *A3018*.**
